# Isolation and Antimicrobial Susceptibility Patterns of *Campylobacter* Species among Diarrheic Children at Jimma, Ethiopia

**DOI:** 10.1155/2014/560617

**Published:** 2014-01-11

**Authors:** Belay Tafa, Tsegaye Sewunet, Haimanot Tassew, Daniel Asrat

**Affiliations:** ^1^School of Medicine, Dire Dawa University, Dire Dawa, Ethiopia; ^2^Department of Medical Laboratory Sciences and Pathology, College of Public Health and Medical Sciences, Jimma University, Jimma, Ethiopia; ^3^Department of Medical Microbiology, Immunology & Parasitology, College of Allied Health Sciences, Addis Ababa University, Addis Ababa, Ethiopia

## Abstract

*Introduction*. *Campylobacter* is one of the leading bacterial causes of food-borne disease. The prevalence of *Campylobacter* species resistant to antimicrobial agents is increasing. This study is intended to determine prevalence and antimicrobial susceptibility patterns of *Campylobacter* species among under-five children with diarrhea. *Methodology*. A cross-sectional study was conducted among 227 under-five children with diarrhea from July to October 2012 at Jimma town. Isolation and identification of *Campylobacter* species were performed using standard bacteriological techniques. Antimicrobial susceptibility test was performed following standard protocol. Chi-square and Fisher's exact tests were used for analysis. *Results*. From 227 under-five children, 16.7% were positive for *Campylobacter* spp.; isolates, *C. jejuni*, *C. coli*, and *C. lari*, accounted for 71.1%, 21.1%, and 7.9%, respectively. Higher rate of resistance was observed to ampicillin 76.3%, trimethoprim-sulfamethoxazole (68.4%), tetracycline (39.5%), chloramphenicol (31.6%), clindamycin (26.3%), and doxycycline (23.7%). Erythromycin, ciprofloxacin, gentamicin, norfloxacin, and nalidixic acid were effective for more than 80% of the isolates. Multiple drug resistance was observed among 78.9% of all the three spp. *Conclusions*. Isolation rate of *Campylobacter* spp. was high. *C. lari* was reported for the first time at this study area. Higher rate of resistance was observed to the commonly used drugs.

## 1. Introduction

Diarrheal diseases constitute a major burden of disease in the world, especially in low- and middle-income countries. Of all medical conditions, diarrhea is the second leading cause of time lost to illness, 72.8 million disability adjusted life years (DALYs) [1]. Diarrheal illnesses are particularly dangerous for young children who are more susceptible to dehydration and nutritional losses during an episode of acute diarrhea. Around 90% of diarrhea-related deaths occur among under-five children living in low- and middle-income countries. Over 1.8 million under-five children die of diarrheal disease, this accounts for 19% of all childhood deaths [2].

Nowadays, *Campylobacter* is the most common cause of bacterial gastroenteritis in developed and developing countries. It is responsible for 400–500 million cases of diarrhea each year [3]; the number of cases often exceed those of salmonellosis and shigellosis. As the result of an epidemiological study of human health burden of foodborne infections in Japan, the estimated burden of *Campylobacter* infections was the highest among the other pathogens. The estimated incidence per 100,000 per year in this region was 237 cases for *Campylobacter*, 32 cases for *Salmonella*, and 15 cases for *V. parahaemolyticus* [4].

In the European Union, *Campylobacter* infection has been the most commonly reported zoonotic diseases. The EU notification rate was 50.28 per 100,000 populations in 2011. *Campylobacter* continued to be the most commonly reported gastrointestinal bacterial pathogen in humans in the EU since 2005. The number of reported confirmed cases of human campylobacteriosis in the EU was 220,209 in 2011. This shows an increase of 2.2% compared to 2010 [5].

The Foodborne Diseases Active Surveillance Network (FoodNet) of the Centers for Disease Control and Prevention (CDC) in the USA estimated that, in 2009, the number of reported infections and incidence per 100,000 populations by *Campylobacter *was 6,033 and 13.02, respectively. In fact, more than 10,000 cases of campylobacteriosis are reported each year to the CDC (approximately six cases per 100,000 persons in the population). However, many more cases remain undiagnosed or unreported [6].

*Campylobacter *infections are typically self-limited, and severe complications are quite rare. However, case treatment was needed; the failure to administer appropriate antibiotics was associated with fatal outcome in bacteremia caused by *Campylobacter* species. The attribute mortality of *Campylobacter* bacteremia was estimated to be 4–16% as described by previous studies [7–9]. The study in Finland indicated that, within 30 days of admittance to hospital, 3% of the patients died [10]. Guillain-Barré syndrome, a debilitating autoimmune disorder, can occur subsequent to infection by *Campylobacter jejuni*. Reiter's syndrome, characterized by arthritis, urethritis, and conjunctivitis, may result from or accompany infection by *Campylobacter *[11].

Most of the time, acute gastroenteritis due to *Campylobacter *species will be treated empirically with fluoroquinolones and macrolides. Although the rate of *Campylobacter *spp. resistance to these drugs is increasing in the world, the incidence is higher in developing countries. Use of these drugs for infections other than gastroenteritis and self medications is often the cause of resistance in developing countries. In developed countries, resistance is due to their use in food animals and travel to developing countries [12, 13].

In Ethiopia, few studies reported that *Campylobacter* species are common cause of childhood diarrhea and antimicrobial resistant strains were also reported [14–17]. The absence of national surveillance program, limited routine culture availability for the isolation of *Campylobacter* species at clinical and research settings, and the need for selective media and unique growth atmosphere make it difficult to give an accurate picture of the burden. This fact indicates that *Campylobacter* as a causative agent of diarrhea is not given appropriate weight and consideration in Ethiopia. Therefore, the objective of this study was to investigate the prevalence and antimicrobial susceptibility patterns of *Campylobacter* species among under five-children with diarrhea in Jimma town.

## 2. Methodology

### 2.1. Study Design and Setting

A cross-sectional study was conducted at Jimma University Specialized Hospital (JUSH) and Jimma Health Center (JHC) from July to October 2012 G.C. A total of 227 under-five patients with diarrhea (defined according to WHO) [18] visiting Jimma University Specialized Hospital (101) and Jimma Health Center (126) were consecutively enrolled in the study.

### 2.2. Sample Collection, Isolation, and Identification of *Campylobacter* Species

Data was collected only after the protocol was approved by Jimma University College of Public Health and Medical Sciences Ethical Review Board (JU/CPMS/ERB) and assent was obtained from the guardian and/or care giver. A structured questionnaire was used to collect sociodemographic data, clinical history, and risk factors by attending physician. Freshly passed stool swab specimen was collected and immediately placed in Cary Blair transport medium (Oxoid Ltd., England) and transported to Jimma University Medical Microbiology Laboratory in ice cold box within 4 hours of collection and processed immediately [19].

The stool samples were inoculated on blood free *Campylobacter* selective agar base containing *Campylobacter* selective supplement comprising cefoperazone, amphotericin B, and teicoplanin (CAT selective supplement; Hi-Media, Mumbai, India) and incubated for 48 hours at 42°C in anaerobic jar (Oxoid Ltd., England) under a microaerophilic atmosphere provided by Gas generating sachets containing 5% O_2_, 10% CO_2_, and 85% N_2_ (Campy-*Gen*; Oxoid Ltd.).

Suspected colonies (greyish, flat, and moistened, with a tendency to spread, having a metal sheen) on selective media were examined for colony morphology. Saline wet mount was performed and microscopically examined for the characteristic darting motility with the iris diaphragm closed to contrast the field. Gram staining was performed to see a gram negative organism with curved or sea-gull wing appearance. Catalase and oxidase tests were performed to confirm the genera* Campylobacter. *The suspected colonies were subcultured on blood agar plates, containing 5% sheep blood, and incubated under microaerophilic conditions at 37°C for 24 hours. Suspected colonies on blood agar were noted for absence of hemolysis, shiny, convex, and colorless to grayish colony characteristics with irregular or round edged nature. The organisms were identified to species level by hippurate hydrolysis and susceptibility to cephalothin (30 *μ*g) and nalidixic acid (30 *μ*g) [20, 21].

### 2.3. Antimicrobial Susceptibility Testing

Antimicrobial susceptibility pattern of the strains isolated was determined by using Kirby-Bauer disk diffusion technique. Mullen-Hinton agar supplemented with 5% sheep blood was prepared. A 0.5 McFarland turbidity standard equivalent bacteria suspension for inoculation was prepared and inoculated. Antimicrobial disks were applied and the plate was incubated at microaerophilic atmospheric condition, at 37°C for 48 hours.

The following antimicrobials were used with their respective concentrations in parenthesis: Ampicillin (AMP, 10 *μ*g), chloramphenicol (C, 30 *μ*g), trimethoprim-sulfamethoxazole (SXT, 25 *μ*g), erythromycin (E, 15 *μ*g), clindamycin (DA, 2 *μ*g), gentamicin (CN, 10 *μ*g), ciprofloxacin (CIP, 5 *μ*g), norfloxacin (NOR, 10 *μ*g), doxycycline (DO, 30 *μ*g), tetracycline (TE, 30 *μ*g), cephalothin (KF, 30 *μ*g), and nalidixic acid (NA, 30 *μ*g). All the discs were from Oxoid Ltd. company, England, UK.

After 48 hours of incubation, the inhibition zones were measured to the nearest millimeter using a graduated ruler. The diameters of inhibition zones were measured around the disks and interpreted on the bases of CLSI 2011 interpretive criteria for Enterobacteriaceae to classify as sensitive, intermediate, or resistant [22] as described by others [23, 24].

*Campylobacter jejuni* (ATCC 700819), *Campylobacter coli* (ATCC 33559), *Staphylococcus aureus* (ATCC 25923), *Enterococcus faecalis* (ATCC 29212), *Pseudomonas aeruginosa* (ATCC 27853), and *E.coli* (ATCC 25922) were used as control strains.

### 2.4. Statistical Analysis

Data were cleaned and entered into a computer and statistical analysis was performed using SPSS version 16. The study findings were explained in tables. Chi-square and Fisher's exact tests were used to test the differences between proportions, and *P* value less than 0.05 was considered statistically significant.

## 3. Results

### 3.1. Age and Sex Distribution of Study Subjects

Of the total 227 under-five children who participated in the study, 121 (53.3%) were females and 106 (46.7%) were males. The mean age of the children was 37.88 (±15.961) months (ranges from one to 59 months). Among the participants, 116 (51.1%) were in the age group of 48–59 months followed by the age group of 36–47 months which accounted for 35 (15.4%). The age group of 12–23 and 24–35 months accounted for 31 (13.7%) each, while the least 14 (6.2%) were in the category of less than 12 months.

### 3.2. Isolation Rate of *Campylobacter* Species from Study Subjects

*Campylobacter* species were isolated from 38 (16.7%) of the total 227 diarrheic under five-children. Among the isolates, 27 (71.1%), 8 (21.1%), and 3 (7.9%) were *C. jejuni*, *C. coli*, and *C. lari*, respectively. Isolation rate of *Campylobacter* species was not statistically different between male and female subjects as well as within the age groups (*P* > 0.05) (Table 1). However, the isolation rate of *Campylobacter *spp. was higher among children of < 2 years old (31.1% as compared to children of age ≥2 years old (13.2%) with statistically significant difference (*P* = 0.008). *Campylobacter *infection had statistically significant association with presence of fever, consistency of stool, and contact with domestic animals (*P* < 0.05). However, the other clinical presentations and risk factors showed no statistically significant associations with *Campylobacter* infection (*P* > 0.05) (Table 2).

### 3.3. Antimicrobial Susceptibility Pattern of *Campylobacter* Isolates

Antimicrobial susceptibility tests were performed for all *Campylobacter *strains isolated. Twelve antimicrobial agents were tested by disk diffusion method. Among the isolates, higher rate of resistant isolates was observed for ampicillin (76.3%), trimethoprim-sulfamethoxazole (68.4%), tetracycline (39.5%), chloramphenicol (31.6%), clindamycin (26.3%), and doxycycline (23.7%). The results are summarized in Table 3. Multiple drug resistance to two or more antimicrobial agents was observed in 78.9% (30/38) of *Campylobacter* isolates. Cephalothin was not considered as a multiple resistant variant property as most campylobacters are inherently resistant to this agent.

## 4. Discussion

The current study revealed the overall prevalence of *Campylobacter *spp. among diarrheic under-five children was 16.7% in Jimma town. When compared with previous reports from Ethiopia, the prevalence is comparable to some of the studies; Gondor teaching hospital, 13.8% [17] and Addis Ababa, 13.7% [16], and it is relatively higher as compared to Dembia district of Gondor, 10.5% [15] and Bahir Dar, 8% [25]. This variation might be attributed to palpable demographic, geographic, and study period differences between these studies. The present finding (16.7%) is also higher than that of the previous report from Jimma, 11.6% [14]. The differences in age groups and/or study periods may also be the possible explanations for the variation of the results.

On the other hand, lower rates of isolation were reported from some other African countries like Uganda 9.3% [26], Madagascar 9.7% [27], and Mozambique 1.7% [28]. Compared with the report from Vhembe district of South Africa, 24.9% [24], the isolation rate of the current study is lower. This might be because Samie and colleagues used filtration technique which facilitates the isolation of antibiotics sensitive *Campylobacter* strains due to the absence of antibiotics cocktail.

As the result of phenotypic characterizations, three different *Campylobacter *spp., *C. jejuni* (71.1%), *C. coli* (21.1%), and *C. lari* (7.9%), were identified in the current study. Of the limited number of studies conducted in Ethiopia on human subjects, only few characterized *Campylobacter* at species level. The species distribution of the present study is different from that of the previous studies [16, 25] where the *C. lari* was not previously identified. On the other hand, the species distribution of *Campylobacter* in the current study is similar with that of the study from Uganda where *C. jejuni*, *C. coli*, and *C. lari* were reported [26].

In the current study, *Campylobacter *spp. isolation was the highest (35.7%) in the age group of less than 12 months followed by the group of 12–23 months (29%) and the lowest (9.7%) in the age group of 24–35 months. This proportional difference within age groups was not statistically significant. This is in agreement with the studies conducted on similar age groups in Mozambique and Uganda [26, 28]. However, culture positivity for *Campylobacter *spp. in children younger than 2 years old was higher than in those greater than or equal to 2 years of age. And the difference is statistically significant. In tropical developing countries, *Campylobacter* was the most commonly isolated pathogen from children less than 2 years old [29, 30].

Clinical findings such as tenesmus, vomiting, and abdominal pain were not statistically associated with culture positivity for *Campylobacter *spp. as it is also reported by other studies [14, 15], whereas fever was significantly associated. This study revealed that there was statistically significant association between *Campylobacter* infection and consistency of stool as reported by Beyene and his colleague [14]. Though it is difficult to diagnose *Campylobacter* based on clinical symptoms, fever and bloody stools may be suggestive to campylobacteriosis. Consuming raw milk, drinking water from unprotected source, and contact with diarrheic person were not statistically associated with culture positivity for *Campylobacter *spp.

On the other hand, there was statistically significant association between contact with domestic animals and recovery rate of *Campylobacter *spp. We indicate that domestic animals such as pets, cattle, sheep, and chickens may play a role for *Campylobacter* infection as described earlier [14, 31].

All *Campylobacter* strains isolated in the current study were resistant to cephalothin as most of these species are inherently resistant to the drug. In this study, higher resistance of *Campylobacter* isolates was recorded to ampicillin (76.3%), trimethoprim-sulfamethoxazole (68.4%), tetracycline (39.5%), chloramphenicol (31.6%), clindamycin (26.3%), and doxycycline (23.7%). Studies in Ethiopia and elsewhere on humans showed that resistance to ampicillin was 18.8% [25] and 20% [26]. A study by Beyene (2004) in the area also showed a lower resistance to ampicillin 50% [14]. A previous study from Addis Ababa, Ethiopia [32], showed that resistance of *Campylobacter* stains to ampicillin was 60% while it was 58.8% for trimethoprim-sulfamethoxazole indicating a slightly lower resistance as compared with the result of the present study. In contrast to the current study, higher resistance to ampicillin, 87% [33] and 100% [34], was reported. Slightly comparable resistance was reported to trimethoprim-sulfamethoxazole, 60% [14], to the result of our study (68.4%). A lower resistance to tetracycline, 14%, 22%, and 22.2%, was reported by others [14, 25, 28] while a higher resistance, 64%, was reported from Iran [34]. However, slightly comparable resistance was reported to tetracycline, 35%, from South Africa [33] to our study (39.5%). None of the strains were resistant to chloramphenicol from the previous report [14], unlike the resistance to chloramphenicol from our finding (31.6%). A lower resistance to chloramphenicol, 11%, was reported [28] as compared with the present findings. Unlike the present study, all *Campylobacter* strains were sensitive to chloramphenicol, erythromycin, gentamicin, nalidixic acid, norfloxacin, and tetracycline as reported from Addis Ababa [32]. The high percentage of strains resistant to ampicillin, trimethoprim- sulphamethoxazole, tetracyclines, and chloramphenicol in this study may be the result of the easy availability of these drugs. Everywhere in Ethiopia, in hospitals and private pharmacies and in the markets, people have easy access to these drugs without prescription. This probably means that the selective pressure of these commonly used antibiotics on the bacteria circulating in the community has resulted in a high frequency of resistance among our isolates.

Resistance to ciprofloxacin and erythromycin, both 12.5% [25], is almost similar to 15.8% and 18.4% of our results to ciprofloxacin and erythromycin, respectively. Similar to our study, (10.5%) resistance to nalidixic acid, 11% resistance rate was observed elsewhere [28]. However, a higher resistance was reported to nalidixic acid from other studies, 54% [33] and 64% [34]. Emergence of resistance to multiple antimicrobial agents in pathogenic bacteria has become a significant public health threat as there are fewer, or even sometimes no, effective antimicrobial agents available for infections caused by these bacteria. In the present study, 78.9% of *Campylobacter* isolates were found multiple resistant to the antimicrobials tested. The high percentage of resistant strains and multiple drug resistant strains of *Campylobacter *spp. to most frequently used antibiotics may be due to uncontrolled use of antibiotics such as self medication and access without prescription [35].

## 5. Conclusions

The prevalence of *Campylobacter* species among under-five children at Jimma town is high. There were three species *C. jejuni, C. coli,* and *C. lari* identified. *C. lari *isthe first to be reported form this study area. These isolates showed higher resistance to commonly prescribed drugs in the area. Isolation of *Campylobacter *spp.was significantly associated with contact with domestic animals among the study subjects which probably transferred resistant strains of the organisms to children through direct contact or environmental contamination. Continuous assessment of the prevalence and antimicrobial susceptibility patterns of *Campylobacter* species in hospitals and in the community is required. Hence, the data may be used as a baseline for large-scale and public health research.

Reducing/avoiding animal contact and judicious use of antimicrobial agents could safeguard antimicrobial efficacy and substantially reduce diarrheal illness caused by *Campylobacter *spp.

## Figures and Tables

**Table 1:** Isolation of *Campylobacter *spp. by sex and age group among under-five children with diarrhea at Jimma University Specialized Hospital (JUSH) and Jimma Health Center (JHC), Jimma, Southwest Ethiopia, July–October 2012.

Variables	Category	Positive		Negative		*P* value
		*N*	%	N	%	
Sex	Male	20	18.9	86	81.1	0.532
	Female	18	14.9	103	85.1	

Age group in months^a^	<12	5	35.7	9	64.3	0.060
	12–23	9	29.0	22	71.0	
	24–35	3	9.7	28	90.3	
	36–47	5	14.3	30	85.7	
	48–59	16	13.8	100	86.2	

^a^Age category is according to the Ethiopia Demographic and Health Survey 2011.

**Table 2:** Isolation of *Campylobacter *spp. by clinical data and risk factors among under-five children with diarrhea at Jimma University Specialized Hospital (JUSH) and Jimma Health Center (JHC), Jimma, Southwest Ethiopia, July–October 2012.

Clinical variables	Isolation of *Campylobacter* spp.				*P* value
	Positive		Negative	
	*N*	%	N	%
Fever					
Yes	31	21.8	111	78.2	0.013^b^
no	7	8.2	78	91.8	
Vomiting					
Yes	19	18.3	85	81.7	0.697
no	19	15.4	104	84.6	
Tenesmus					
Yes	18	15.5	98	84.5	0.744
no	20	18.0	91	82.0	
Abdominal pain					
Yes	35	17.7	163	82.3	0.430^c^
no	3	10.3	26	89.7	
Duration of diarrhea in days					
1–5	31	18.8	134	81.2	0.559
6–10	4	9.5	38	90.5	
11–15	2	18.2	9	81.8	
≥16	1	11.1	8	88.9	
Consistency of stool					
Watery	13	15.7	70	84.3	0.001^b^
Mucoid	14	11.6	107	88.4	
Bloody	9	52.9	8	47.1	
Mixed (mucus and blood)	2	33.3	4	66.7	
Contact with domestic animals					
Yes	25	23.8	80	76.2	0.014^b^
no	13	10.7	109	89.3	
Consuming raw milk					
Yes	4	36.4	7	63.6	0.092^c^
no	34	15.7	182	84.3	
Source of drinking water					
Unprotected	7	26.9	19	73.1	0.162^c^
Protected	31	15.4	170	84.6	
Contact history with diarrheic person					
Yes	1	8.3	11	91.7	0.696^c^
no	37	17.2	178	82.8	

^b^Statistically significant; ^c^Fisher's exact test.

**Table 3:** Antimicrobial susceptibility profile of *Campylobacter* spp. isolated from under-five children with diarrhea at Jimma University Specialized Hospital (JUSH) and Jimma Health Center (JHC), Jimma, Southwest Ethiopia, July–October 2012.

Antimicrobial agents	Number of *Campylobacter* isolates			% of resistant isolates
	S	I	R	
Ampicillin (10 *μ*g)	9	0	29	76.3
Chloramphenicol (30 *μ*g)	26	0	12	31.6
Tetracycline (30 *μ*g)	20	3	15	39.5
Gentamicin (10 *μ*g)	31	2	5	13.2
Doxycycline (30 *μ*g)	26	3	9	23.7
Trimethoprim-sulfamethoxazole (25 *μ*g)	12	0	26	68.4
Ciprofloxacin (5 *μ*g)	30	2	6	15.8
Norfloxacin (10 *μ*g)	34	0	4	10.5
Erythromycin (15 *μ*g )	29	2	7	18.4
Clindamycin (2 *μ*g)	24	4	10	26.3
Cephalothin (30 *μ*g)	0	0	38	100
Nalidixic acid (30 *μ*g)	34	0	4	10.5

Keys: S: sensitive; I: intermediate; R: resistant.
